# Erythrocyte Adhesion of Merozoite Surface Antigen 2c1 Expressed During Extracellular Stages of *Babesia orientalis*

**DOI:** 10.3389/fimmu.2021.623492

**Published:** 2021-05-17

**Authors:** Zheng Nie, Yangsiqi Ao, Sen Wang, Xiang Shu, Muxiao Li, Xueyan Zhan, Long Yu, Xiaomeng An, Yali Sun, Jiaying Guo, Yangnan Zhao, Lan He, Junlong Zhao

**Affiliations:** ^1^ State Key Laboratory of Agricultural Microbiology, College of Veterinary Medicine, Huazhong Agricultural University, Wuhan, China; ^2^ Key Laboratory of Development of Veterinary Diagnostic Products, Ministry of Agriculture of the People’s Republic of China, Wuhan, China; ^3^ Key Laboratory of Preventive Veterinary Medicine in Hubei Province, The Cooperative Innovation Center for Sustainable Pig Production, Wuhan, China

**Keywords:** invasion, adhesion, MSA-2c1, *Babesia orientalis*, merozoite surface antigen

## Abstract

*Babesia orientalis*, a major infectious agent of water buffalo hemolytic babesiosis, is transmitted by *Rhipicephalus haemaphysaloides*. However, no effective vaccine is available. Essential antigens that are involved in parasite invasion of host red blood cells (RBCs) are potential vaccine candidates. Therefore, the identification and the conduction of functional studies of essential antigens are highly desirable. Here, we evaluated the function of *B. orientalis* merozoite surface antigen 2c1 (BoMSA-2c1), which belongs to the variable merozoite surface antigen (VMSA) family in *B. orientalis*. We developed a polyclonal antiserum against the purified recombinant (r)BoMSA-2c1 protein. Immunofluorescence staining results showed that BoMSA-2c1 was expressed only on extracellular merozoites, whereas the antigen was undetectable in intracellular parasites. RBC binding assays suggested that BoMSA-2c1 specifically bound to buffalo erythrocytes. Cytoadherence assays using a eukaryotic expression system *in vitro* further verified the binding and inhibitory ability of BoMSA-2c1. We found that BoMSA-2c1 with a GPI domain was expressed on the surface of HEK293T cells that bound to water buffalo RBCs, and that the anti-rBoMSA2c1 antibody inhibited this binding. These results indicated that BoMSA-2c1 was involved in mediating initial binding to host erythrocytes of *B. orientalis.* Identification of the occurrence of binding early in the invasion process may facilitate understanding of the growth characteristics, and may help in formulating strategies for the prevention and control of this parasite.

## Introduction

Parasites belonging to the genus *Babesia* are tick-borne intraerythrocytic protozoans that belong to the phylum Apicomplexa. This pathogen manifests in Pan America, Europe, Africa, and Asia (1, 2). Over 100 species of *Babesia* parasites reportedly infect vertebrates and birds worldwide and their distribution corresponds to that of their tick vector. Additionally, infection in the wild is commonly asymptomatic, and farm, companion animals, and pets can develop typical clinical signs such as fever, anemia, jaundice, and hemoglobinuria (3).

Similar to the characteristics observed in other hemoparasites, extracellular merozoites exhibit reversible attachment to host RBCs; furthermore, reorientation of the merozoite enables organization of the apical organelles close to the attachment interface for the formation of tight junctions. During invasion, the RBC membrane undergoes invagination and forms a parasitophorous vacuole (PV) (4). After completion of a parasitic invasion of erythrocytes, the PV disintegrates and resides freely within the host RBC cytoplasm. Parasites produce two merozoites by binary fission. After the occurrence of erythrocyte lysis, merozoites are released and the invasion of new RBCs occurs (5, 6). This aspect of the life cycle is the main reason for the development of anemia and hemoglobinuria that commonly occurs in infected animals. Therefore, focus has been directed toward the prevention and control of *Babesia* by blocking this process. At the initial step of erythrocyte invasion, *Babesia* parasites reportedly utilize glycosyl phosphatidyl inositol (GPI) anchor antigens to enable attachment to target cells (7). However, GPI-anchored antigens are often highly variable surface proteins and are not conserved between *Babesia* species (8). The GPI-anchored antigens frequently contain unique domains such as variable merozoite surface antigen (VMSA) (*Babesia bovis* and *Babesia orientalis*) (9), Bc28 (*Babesia canis*) (10), and Bd37 (*Babesia divergens*) (11). Regardless of the *Babesia* species, the role of GPI-anchored proteins in the early step of cell invasion involves their exposure to the extracellular environment (12). Such a phenomenon of localization during the extracellular step of the parasite life cycle helps in the consideration of the GPI-anchored proteins as antigens that are potentially recognized by antibodies or other immune effectors (13, 14). Consequently, this type of parasitic proteins is considered among the most promising candidates for the development of an effective vaccine (15, 16).

*Babesia orientalis* causes water buffalo babesiosis which is endemic to central and southern China, and its infection rates are much higher than those of *B. bovis* and *B. bigemina* (17). This parasite is transovarially transmitted by *Rhipicephalus haemaphysaloides* which is the only known vector of *B. orientalis*, and water buffalo is the only known host (18). The GPI-anchored proteins in *B. orientalis* include BoMSA-1, -2a1, -2a2, -2c1, and -2c2. Characterization of these proteins has revealed the presence of a GPI anchor domain at the C-terminal, and a signal peptide at the N-terminal (19). The proteins transcribed by members of this gene family are hypothesized to harbor B-cell epitopes (19). Characterization, combined with sequencing results, identified BoMSA-2c1 as the smallest protein containing the least B-cell epitopes, indicating that it might play an important role in the survival of *B. orientalis* (19). A proline-rich hypervariable region (HVR) has been predicted in the BoVMSA family, similar to that observed in the BbVMSA family in *B. bovis*, where proline-rich regions in other apicomplexan surface regions may be involved in host cell binding (19–22). To bridge the knowledge gaps regarding adhesion factors during *B. orientalis* invasion, we aimed to determine the molecular function of BoMSA-2c1. Our findings provide insights into the involvement of this GPI-anchored antigen in adhesion of *B. orientalis* merozoites to host RBCs suggesting an importance for host cell invasion.

## MATERIALS AND METHODS

### Gene Cloning and Nucleotide Sequencing of BoMSA-2c1

The gene fragment encoding the BoMSA-2c1 extracellular region corresponding to nucleotide G^70^ to the end (A^870^) was amplified using PCR. The forward (MSA-2c1F: 5′-*ACC GCG AAC AGA TTG GAG GT*G CTA CGA CGC CTC GTT TG-3′) and reverse (MSA-2c1R: 5′-*TCG AAT TCG GAT CCT CTA GT*T TAA AAT GCA GAG AGA ACA ATG TAG C-3′) primers contained the homologous arm from the plasmid pE-sumo (underlined in italics). The vector was linearized by PCR using the primers (pE-sumo F: 5′-ACT AGA GGA TCC GAA TTC GA-3′; and pE-sumo R: 5′-ACC TCC AAT CTG TTC GCG GT-3′). The PCR products were subjected to digestion, and then, the region of interest was purified and cloned using the ClonExpress™ II One Step Cloning Kits (Vazyme Biotech Co., Nanjing, China). The BoMSA-2c1 was then cloned into the expression vector pE-sumo, which possesses a 6×His-sumo tag to facilitate purification. The purified expression vector was transformed into One Shot™ BL21 Star™ (DE3) (TransGen Biotech Co., Ltd., Beijing, China) competent *E. coli* cells. The recombinant protein was expressed after induction with 0.08 mM isopropyl-β-d-thiogalactopyranoside (IPTG) for 12 h at 28°C. The supernatant was loaded onto preequilibrated Ni^2+^-nitrilotriacetic acid (NTA) agarose columns and subjected to shaking conditions at 4°C for 3 h as per the manufacturer’s instructions. The expression and purification of recombinant proteins were assessed by performing 12% sodium dodecyl sulfate polyacrylamide gel electrophoresis (SDS-PAGE). The concentration of the recombinant protein was measured using BCA protein assay kits (Beyotime Institute of Biotechnology, Nanjing, China).

### Preparation of Anti-rBoMSA-2c1 Immune Serum

Anti-rBoMSA-2c1 sera were developed in New Zealand white rabbits after the collection of pre-immune serum. The rabbits were subcutaneously injected with 500 µg of recombinant BoMSA-2c1 (rBoMSA-2c1) emulsified in Freund’s complete adjuvant (Sigma-Aldrich Corp., St. Louis, MO, USA) on day 0. The same antigen was administered using Freund’s incomplete adjuvant (Sigma-Aldrich Corp.) after 14, 21, and 28 days. Sera were collected when the antibody titers reached 10^6^, which were assayed by an ELISA using the recombinant protein, rBoMSA-2c1. Total immunoglobulin G (IgG) was purified from the sera by performing chromatography using a Protein column A (Beyotime Institute of Biotechnology) as per the manufacturer’s instructions, and serum samples were then stored at −20°C.

### Immunoblotting

To identify the response of *B. orientalis*-infected water buffalo to rBoMSA-2c1, the recombinant protein was resolved by SDS-PAGE, blotted onto nitrocellulose membranes (Merck, KGaA, Darmstadt, Germany), and incubated for 1 h at 37°C in PBS containing 0.1% Tween 20, and 5% skim milk). Duplicate blots were incubated with *B. orientalis-*positive serum (1:500 in PBS, 0.1% Tween 20) or 1:500-diluted normal water buffalo serum (negative control) overnight at 4°C. Both sera were collected during previous animal experiment and stored in −20°C in our lab (23). The membranes were then subjected to probing using 1:2000-diluted secondary antibodies (BovIgG/HRP, Bioss Inc., Woburn, MA, USA) at 37°C for 30 min. The reactions were visualized using direct ECL chemiluminescence (Thermo Fisher Scientific Inc., Waltham, MA, USA).

Lysed erythrocytes (0.5 mg per lane) from *B. orientalis*-infected was extracted from liquid nitrogen stabilizes, *Babesia* free water buffalo (negative control) were resolved by performing 12% SDS-PAGE, and were then blotted onto nitrocellulose membranes (Millipore Sigma Co., Ltd., Burlington, MA, USA) to detect native BoMSA-2c1 in *B. orientalis* merozoites. Nonspecific protein binding was blocked in TBS containing 0.05% Tween-20 (TBST) and 1% BSA overnight at 4°C. The blots were probed with rabbit serum developed against rBoMSA-2c1, or pre-immune serum diluted (1:500) with TBST for 3 h at 37°C. The blots were finally incubated with secondary goat anti-rabbit IgG antibody conjugated to horseradish peroxidase (Amersham Pharmacia Biotech, Little Chalfont, UK) diluted 1:2000 with TBST at 37°C for 1 h. Positive blots were visualized using ECL.

### Indirect Immunofluorescence Assay (IFA)

Blood smears on slides were prepared from previous animal experiments and stored at −20°C in our lab (24). The slides were fixed in cold methanol for 1 min. Nonspecific protein binding was blocked after overnight incubation using PBS (pH 7.2) containing 10% FBS. The slides were washed three times with PBS, and then indirectly stained for immunofluorescence using anti-rBoMSA-2c1–specific IgG that was diluted 1:100 with 1× PBS; results were observed after incubation at 37°C for 1 h. The secondary antibody used was Alexa Fluor 488-conjugated goat anti-rabbit IgG (Life Technologies, Inc., Rockville, MD, USA) diluted 1:1000 with 1× PBS; then, parasite nuclei were visualized by incubation with Hoechst stain for 1 h at 37°C followed by confocal microscopy (Olympus Life Science, Tokyo, Japan).

### Erythrocyte Binding Assays

Erythrocyte binding assay was conducted as follows (25). Water-buffalo erythrocytes (50-µL packed cells) were subjected to washing steps three times with PBS and rotary-incubated with purified recombinant rBoMSA-2c1 (0.5 mg in 1 mL of PBS) for 1 h. Erythrocytes were harvested by centrifugation (1800 × *g*, 3 min) and suspended in 200 μL of PBS. Suspensions were subsequently layered on silicon oil (d = 1005, Sigma-Aldrich Corp.) and sedimented by centrifugation at 4000 × *g* for 3 min. The supernatant and oil were removed, and bound proteins were eluted from erythrocytes using 50 µL of PBS containing 0.5 M NaCl. The bound recombinant MSA-2c1 protein (1.6, 1.2, 0.8, 0.4, 0.2, and 0.1 mg/mL in PBS) was detected by western blotting using an anti-His-Tag monoclonal antibody (Qiagen GmbH, Hilden, Germany).

### HEK 293T Cell Culture and Transfection

HEK 293T cells were cultured in the RPMI-1640 medium (Gibco BRL, Gaithersburg, MD, USA) supplemented with 10% heat-inactivated fetal bovine serum (FBS, Gibco), 2 mM L-Glutamine (Invitrogen), and 2 mM penicillin-streptomycin (Invitrogen, Carlsbad, CA, USA) in a humidified 5% CO_2_ atmosphere at 37°C.

The full length of the BoMSA-2c1 coding region without the stop codon was cloned into the pEGFP-N1 vector using the ClonExpress™ II One Step Cloning Kit (Vazyme). The inserted fragment and the vector were amplified using the following primers: In-BoMSA2c1-F: 5′-*CGG ACT CAG ATC TCG AGC TC*A TGA TGG GTG CTA AGC TTG-3′ and In-BoMSA2c1-R: 5′- *ATG GTG GCG ACC GGT GGA TC*A AAT GCA GAG AGA ACA ATG TAG C-3′; Ve-pEGFPN1-F: 5′-GAT CCA CCG GTC GCC ACC AT-3′ and Ve-pEGFPN1-R: 5′-GAG CTC GAG ATC TGA GTC CG-3, respectively.

Cells were obtained after detachment using trypsin-EDTA (Invitrogen), and suspended to a density of 1 × 10^6^ in 60 μL of EL buffer. The plasmid construct was added to a final concentration of 1 ng/60 μL. The mixture (60 μL/well) was loaded into standard 96-well plates (Corning Inc., Corning, NY, USA), and then the cells were transfected using an H1 electroporator (Suzhou Etta Biotech Co. Ltd., Suzhou, China) as per the manufacturer’s instructions (26). Thereafter, 20 μL/well of transfected cells were transferred into 24-well plates and incubated with 600 μL of the culture medium for 24 h. Cells expressing GFP were evaluated in five randomly selected areas using the IX-71a fluorescence microscope (Olympus Corporation, Tokyo, Japan). Green fluorescent protein (GFP)-tagged cells were assessed in an unfixed manner using the Flowview^®^ FV1000 Laser Scanning Confocal Imaging System (Olympus) at a magnification of 200× and results were scored to determine transfection efficiency.

### Rosette Assays of BoMSA-2c1 Adhesion and Inhibition

We incubated HEK 293T cells 24 h post transfection with buffalo erythrocytes (10% hematocrit) for 2 h at 37°C. The cells were subjected to washing steps with PBS to remove non-adherent RBCs; then, clusters of ≥ 5 RBCs bound to the transfected HEK 293T cells were scored as rosettes in 10 consecutive fields at 200x magnification. Data were normalized at 50% transfection efficiency. All cells transfected with this construct emitted green fluorescence and transfection efficiency was determined as the ratio of fluorescent cells to the total number of cells per 1,000 counts. Negative control cells were transfected with the empty pEGFP-N1 vector (GFP) and non-transfected HEK 293T cells (NT).

We assessed the inhibition of binding by using anti-serum as follows. Rabbit anti-rBoMSA-2c1 sera were diluted to 1:10 (500 µg/mL) and 1:100 (50 µg/mL) using complete RPMI, and then incubated with HEK 293T cells transfected with BoMSA-2c1 (1 mL/well) for 1 h at 37°C with intermittent shaking. Pre-immune serum diluted 1:10 (500 µg/mL) was considered as the control. The HEK 293T cells were subjected to washing steps using incomplete RPMI medium, and binding was assessed as per methods described above. Each condition was assessed in triplicate and the experiment was repeated three times. The numbers of rosettes were examined *via* two-way analysis of variance (ANOVA), followed by Tukey’s multiple-comparison tests. All data were statistically analyzed using GraphPad Prism 7 (GraphPad Software Inc., San Diego, CA, USA). The results are shown as mean ± SD (NS; P > 0.05 not significant). Values were considered statistically significant at P < 0.05.

## Results

### Cloning, Expression, and Purification of rBoMSA-2c1

**Figure 1:**
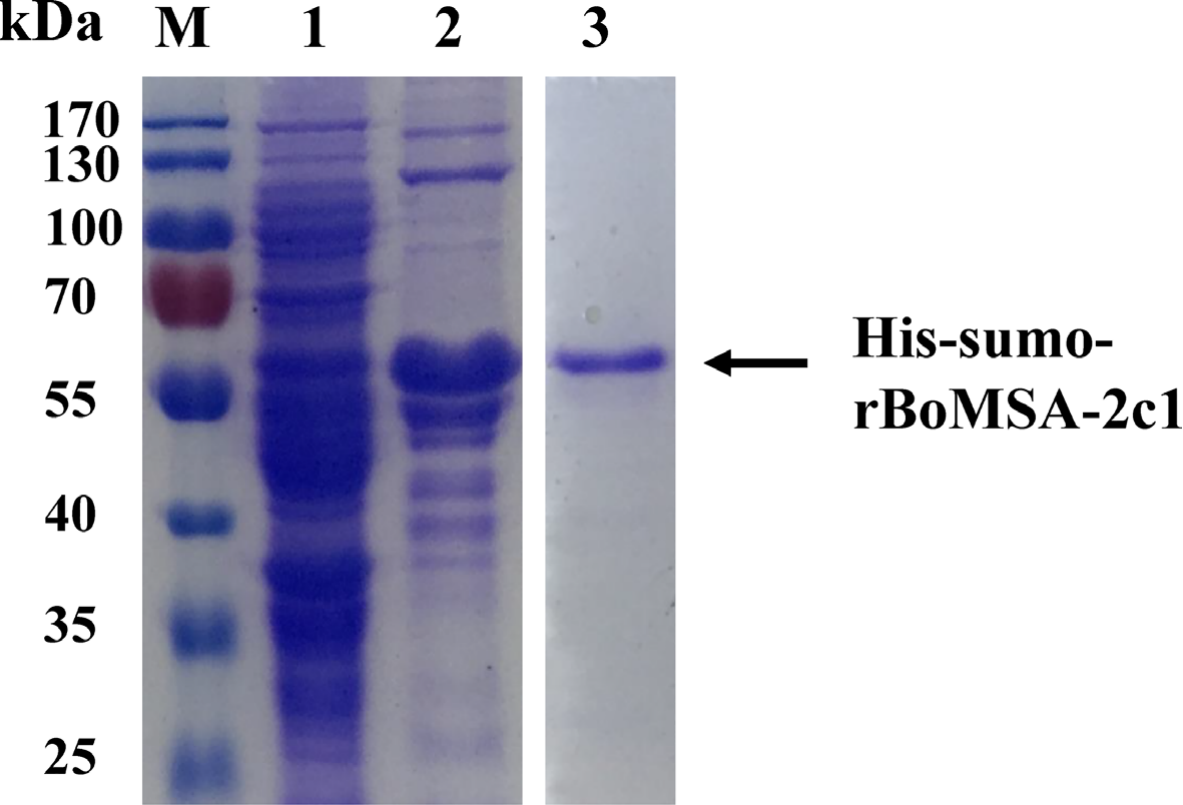
Expression and purification of rBoMSA-2c1. Expression of rBoMSA-2c1 in E. coli BL21 (DE3) assessed by SDS-PAGE. Lysates of pE-sumo-BoMSA-2c1 without (lane 1) and with (lane 2) IPTG induction. Purified product of rBoMSA-2c1 (lane 3). Arrows indicate the corresponding bands. IPTG, isopropyl-β-d-thiogalactopyranoside.

The BoMSA-2c1 gene without the signal peptide sequence was amplified from cDNA using MSA-2c1F and MSA-2c1R primers, because complete BoMSA-2c1 could not be expressed in *E. coli* BL21 (DE3). The predicted molecular weight of this protein fused with the His-SUMO tag was found to be ~48 kDa. Cloned rBoMSA-2c1 was expressed as a ~55-kDa soluble His-SUMO fusion protein. The HVR regions were proline-rich (ck). The rBoMSA-2c1 protein was purified by using the Protein Pure Ni-NTA Resin and was stored at −80°C (**Figure 1**).

### Identification of the Recombinant and Native BoMSA-2c1

**Figure 2:**
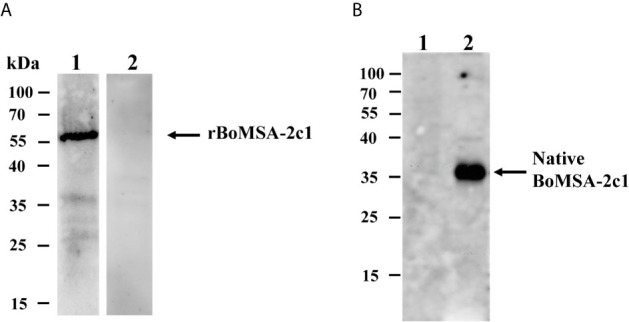
Western blots of BoMSA-2c1. **(A)** Recombinant BoMSA-2c1 subjected to probing with water buffalo serum infected with (lane 1) and without (lane 2) *B orientalis*. **(B)** Immunoblots of the native BoMSA-2c1 protein in parasite lysates. Lane 1, lysate of the uninfected buffalo erythrocytes does not exhibit reaction with polyclonal BoMSA-2c1 antibody. Lane 2, a specific 37-kDa band in parasite lysate subjected to probing with rBoMSA-2c1 antiserum.

The specific antigenicity of BoMSA-2c1 was analyzed by conducting blotting experiments of rBoMSA-2c1 with serum samples collected from a water buffalo infected with *B. orientalis*. The negative control comprised serum obtained from healthy water buffalo. A specific 55-kDa band was detected in positive serum samples, but not in negative serum samples (**Figure 2A**). These results indicated that the MSA-2c1 was a specific antigenic protein.

Native BoMSA-2c1 was identified by performing western blotting against anti-rBoMSA-2c1 rabbit serum. Only anti-rBoMSA-2c1 serum exhibited reaction with the *B. orientalis* lysates and generated a specific 35-kDa band, which was consistent with the predicted molecular weight of mature BoMSA-2c1 (**Figure 2B**). Negative control anti-rBoMSA-2c1 serum subjected to probing with water buffalo ghost erythrocytes and pre-immune serum with *B. orientalis* lysates did not generate any bands.

### Localization of BoMSA-2c1

**Figure 3:**
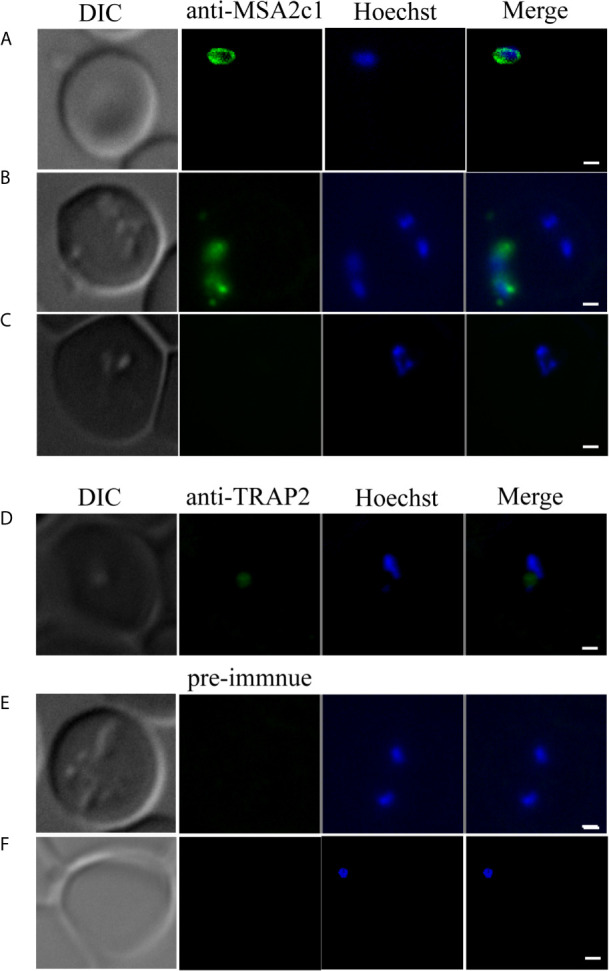
Location of BoMSA-2c1 in *B orientalis* determined by immunofluorescence staining. BoMSA-2c1 is located only on the extracellular parasite membrane. Reactivity of anti-rBoMSA-2c1 serum with parasite during invasion **(A)**, early-egress **(B)**, and intracellular **(C)** stages. Anti-TRAP2 has been used as the positive control for the intracellular parasite **(D)**, and pre-immune serum has been used as the negative control for validation of intracellular and extracellular parasites **(E, F)**. PcAb-BoMSA-2c1 and PcAb-BoTRAP2 (blue) exhibited reactions with native proteins on merozoites. Nuclei are indicated in blue with Hoechst counterstain. Scale bars: 1 μm.

BoMSA-2c1 localization was investigated in *B. orientalis* by performing indirect fluorescent antibody tests (IFAT) using anti-rBoMSA-2c1 immune serum. Free parasites located outside RBCs emitted BoMSA-2c1 signals (**Figures 3A, B** **)**. Fluorescence was undetectable throughout the intracellular merozoite stage (**Figures 3B, C** **)**. Positive control BoTRAP2 was detected at the apical end of the intracellular merozoites as per previously described results (**Figure 3D**) (24). Signals were undetectable when pre-immune serum was included (**Figures 3E, F** **)**.

### BoMSA-2c1 Is an Erythrocyte-Binding Protein

**Figure 4:**
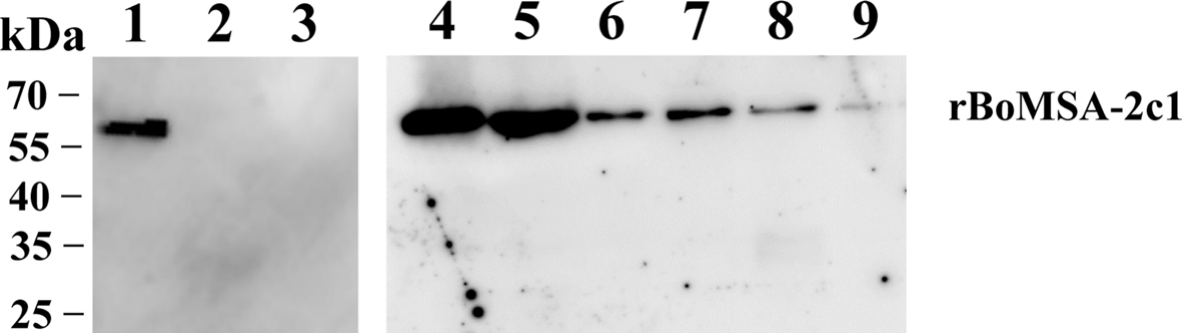
Western blots of RBC binding to rBoMSA-2c1. Water buffalo RBCs (100 µL) binding to 1.0 mg/mL (lane 1) and (lane 2) 0 mg/mL of rBoMSA-2c1, and His-sumo (lane 3). Lanes 4–9, buffalo RBCs binding to rBoMSA-2c1 (1.6, 1.2, 0.8, 0.6, 0.4, and 0.2 mg/mL), respectively. His-sumo has been used as the negative control. All lanes exhibited reactions with anti-His antibodies.

The localization of the BoMSA-2c1 protein suggested that it might play an important role in establishing interactions with erythrocytes. We therefore performed assays to detect rBoMSA-2c1 binding to water buffalo erythrocytes. **Figure 4** showed the binding between purified rBoMSA-2c1 and the water buffalo erythrocytes. Signals increased as the rBoMSA-2c1 concentration increased. The binding was maximal at 1.6 mg/mL of rBoMSA-2c1 and bands were undetectable in the negative control.

### Anti-rBoMSA-2c1 Specifically Inhibits BoMSA-2c1 Binding to Water Buffalo RBCs

**Figure 5:**
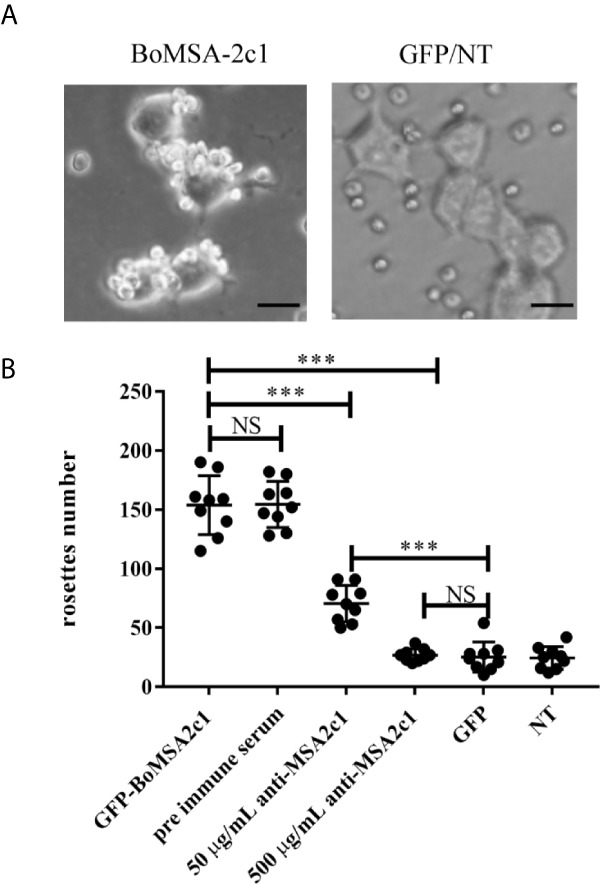
Rosette assays of binding and inhibition of BoMSA-2c1. **(A)** Rosettes formed by HEK 293T cells transfected with BoMSA-2c1. Positive reaction has been defined as the observation of coverage of > 50% of the transfected cell surface with erythrocytes. Negative controls include empty pEGFP-NI vector/non-transfected cells (GFP/NT). **(B)** Numbers of rosettes formed by HEK 293T cells transfected with genes encoding BoMSA-2c1. Positive reaction has been defined as the clustering of ≥ 5 RBCs with the transfected cells, and rosettes were scored in 10 consecutive fields at 200x magnification in each construct. Cells were subsequently incubated with anti-rBoMSA-2c1 rabbit sera at two dilutions before the addition of water buffalo erythrocytes. Pre-immune rabbit sera diluted 1:100, empty pEGFP-NI vector (GFP), and non-transfected (NT) cells were used as controls. Data were analyzed using one-way analysis of variance (ANOVA), followed by Tukey’s multiple comparison tests. Results are shown as mean ± SD (shown as error bars). NS, not significant at P > 0.05. Significant at ***P < 0.001. Scale bars: 40 µm.

We investigated binding capacity *in vitro* by performing cytoadherence assays in a eukaryotic expression system. We found that BoMSA-2c1-GFP, which contains a GPI domain, was expressed on the surface of HEK 293T cells. This assay can be used to analyze only the binding capacity of BoMSA-2c1, expressed on the surface of transfected HEK 293T cells (**Figure S1**) with erythrocytes. We transfected HEK 293T cells for 24 h with BoMSA-2c1, and then incubated them with water buffalo RBCs for 3 h. Binding capacity was measured and determined as the numbers of rosettes containing ≥ 5 RBCs. **Figure 5A** shows that the buffalo RBCs were bound to the surface of transfected HEK 293T cells, thus forming rosettes. Few RBCs even seemed to be in an upright position on the surface of these cells. In contrast, cells transfected with the pEGFP-N1 blank vector or non-transfected (NT) cells did not bind to RBCs, confirming that RBC binding was dependent on BoMSA-2c1 expression.

We explored whether BoMSA-2c1 cytoadherence could be inhibited with the application of its specific antibody. We incubated HEK 293T cells with purified anti-BoMSA-2c1 IgG for 1 h at 37°C, and subjected the cells to washing steps using PBS; then, they were incubated with buffalo RBCs. Pre-immune serum, GFP, and NT groups were considered as the negative controls. The number of rosettes decreased by 51% and almost reached 100% when the cells were incubated with 50 and 500 µg/mL rBoMSA-2c1 antiserum (**Figure 5B**).

## Discussion

We explored the function of rBoMSA-2c1 purified as a soluble protein in *E. coli* BL21 (DE3). This recombinant protein was detected using *B. orientalis*-positive serum, and the use of rBoMSA-2c1 antiserum resulted in the detection of a unique band at ∼37 kDa in parasite lysates. Sizes of the native and recombinant BoMSA-2c1 were greater than the predicted size, which is a common phenomenon observed for the GPI-anchor family (MSA-1) that has been reported in *B. bovis* (27). The molecular weight of BoSBP4, BoRON4, and BoP53 in *B. orientalis* was also greater than the predicted values (28–30).

The VMSA GPI-anchored protein family in *Babesia* shares the same MSA-2c family functional domain reported in *B. orientalis* and *B. bovis*. The VMSA family of proteins in *B. bovis* is expressed on the merozoite and sporozoite surface, and antibodies used against them help neutralize erythrocyte invasion *in vitro* (8, 9). Thus, the VMSA proteins may be considered as vaccine candidates for *B. bovis* (1). The VMSA family members BoMSA-1, BoMSA-2a1, BoMSA-2a2, BoMSA-2c1, and BoMSA-2c2 in *B. orientalis* share the same nucleotide sequences at the C- and N-terminals (19). This phenomenon was considered to be involved in the generation of mutations and insertions/deletions after the occurrence of homologous recombination during the sexual stages of the parasite in the tick vector (8). These five proteins contain unique or shared B-cell epitopes (19). Thus, this family is thought to be involved in the immune evasion of *B. orientalis* (19). However, the molecular function of these proteins remains unknown. The IFAT results of the present study showed that BoMSA-2c1 was expressed only at the extracellular stage of *B. orientalis*, and this finding was consistent with the localization of merozoite surface antigens (MSA) in *Plasmodium falciparum*. The shedding of GPI-anchored proteins seems to be a more widespread phenomenon (31). Before the occurrence of invasion, merozoite surface protein 1 (MSP-1) is cleaved by subtilisin 2 (SUB2) protease (32), and MSP-6 and MSP-7 as part of the MSP-1 complex are shed in this manner (33, 34). The PfMSP2 antibody could perform uniform labeling of the parasites throughout invasion, but immediately underwent degradation within 10 min after invasion (35). Although shedding of peripheral surface proteins occurs likely due to the cleavage and subsequent shedding of membrane-bound ligands of these proteins or degradation *via* an unknown mechanism, all proteins showed binding to RBCs with or without receptors (36).

We investigated the function of BoMSA-2c1 by enabling its expression on the surface of HEK 293T cells through its GPI domain. We found that water buffalo RBCs formed rosettes on the surface of HEK 293T cells, unlike control cells transfected with the empty pEGFP-N1 vector and non-transfected HEK 293T cells (significantly different at P< 0.001). The use of anti-rBoMSA-2c1 antisera inhibited binding of healthy host erythrocytes to HEK 293T cells. These results suggested that BoMSA-2c1 facilitated parasite adhesion to the surface of water buffalo RBCs. Notably, rBoMSA-2c1 without an N-terminal signal peptide can bind to RBCs, and antibodies against this recombinant protein can block the formation of rosettes with the full-length protein, indicating that the N-terminal signal peptide of BoMSA-2c1 is not necessary for binding. To directly verify the surface localization and adhesion function of BoMSA-2c1, conduction of live IFA and a neutralization assays *in vitro* would be necessary. However, due to the death of the donor animal we could not establish a culture of *B. orientalis* based on erythrocytes of this animal preventing the execution of both these assays. Although we have tested RBCs and sera of several other water buffalos these results remained unsuccessful.

Proteolytic shedding of surface adhesins is a common feature of apicomplexan parasites. However, the complexity and properties of this pathway differ among species (37). Other than the occurrence of protein shedding by subtilisins (SUBs) in *Plasmodium*, a family of serine proteinases (ROMs) cleave numerous adhesin proteins in *Toxoplasma gondii* (37). This protein shedding is necessary for productive invasion, and may be important to establish adhesive interactions between the parasite and host cell membrane to facilitate unimpeded passage into nascent PVs (35). The SUB and ROM gene families have been identified in *Babesia* (38, 39); thus, future studies should address the shedding or cleavage of BoMSA-2c1.

We identified BoMSA-2c1 as an extracellular antigen that could be only expressed on parasites outside RBCs. This antigen may help the binding between the parasite and the host RBCs, the binding can be blocked with the use of specific anti-MSA-2c1 antibodies. Cleavage or degradation mechanisms might function during *Babesia* invasion, as signals were not evident after the parasite penetrated the RBC. However, this aspect warrants further investigation. The present findings demonstrate involvement of MSA-2c1 in parasite attachment to the host erythrocyte and suggest it represents a potential vaccine candidate.

## Data Availability Statement

The original contributions presented in the study are included in the article/**Supplementary Material**. Further inquiries can be directed to the corresponding author.

## Ethics Statement

This study was approved by the Scientific Ethic Committee of Huazhong Agricultural University (permit number: HZAUMO-2017-040). All mice were handled in accordance with the Animal Ethics Procedures and Guidelines of the People’s Republic of China.

## Author Contributions

ZN, LH, and JZ designed the study and wrote the draft of the manuscript. YA, ML, XZ, and YZ performed the experiments. SW, LY, XA, YS, JG, and XS participated in the data analysis. All authors contributed to the article and approved the submitted version.

## Funding

This study was supported by the National Natural Science Foundation of China (Grant No. 31930108), the National Key Research and Development Program of China (Grant No. 2017YFD0501201), the Fundamental Research Funds for the Central Universities in China (Project: 2662020DKPY016) and the Fundamental Research Funds for the Central University (Grant No. 2662019PY001).

## Conflict of Interest

The authors declare that the research was conducted in the absence of any commercial or financial relationships that could be construed as a potential conflict of interest.

## Supplementary Material

Supplementary Figure 1 Expression of BoMSA-2c1 on the HEK 293T cell surface. **(A)** Expression of green fluorescent protein (GFP) detected by scanning confocal microscopy (LSCM). BoMSA-2c1-GFP was located on the surface of HEK 293T cells transfected with pEGFP-BoMSA2c1-N1, and GFP was detected in the cytoplasm of HEK 293T cells, transfected with the control pEGFP-N1 vector. **(B)** Western blots of BoMSA-2c1 in HEK 293T cells. Lysates of HEK 293T cells transfected with pEGFP-BoMSA-2c1 (lanes 1, 3, and 5) and empty pEGFP-NI vector (lanes 2, 4, and 6). Lysates of negative control HEK 293T cells exhibiting reactions with PcAb-BoMSA-2c1 (lanes 3 and 4), anti-GFP (lanes 5 and 6), and pre-immune serum (lanes 1 and 2). Scale bars: 40 μm.

## References

[1] Suarez CE Alzan HF Silva MG Rathinasamy V Poole WA Cooke BM. Unravelling the Cellular and Molecular Pathogenesis of Bovine Babesiosis: Is the Sky the Limit? Int J Parasitol (2019) 49(2):183–97. 10.1016/j.ijpara.2018.11.002 PMC6988112 30690089

[2] Zimmer AJ Simonsen KA. Babesiosis. In: Statpearls. Treasure Island (FL: StatPearls Publishing LLC (2021). StatPearls Publishing 2021.

[3] Schnittger L Rodriguez AE Florin-Christensen M Morrison DA. Babesia: A World Emerging. Genet Infect Evol (2012) 12(8):1788–809. 10.1016/j.meegid.2012.07.004 22871652

[4] Gohil S Kats LM Sturm A Cooke BM. Recent Insights Into Alteration of Red Blood Cells by Babesia Bovis: Moovin’ Forward. Trends Parasitol (2010) 26(12):591–9. 10.1016/j.pt.2010.06.012 20598944

[5] Dubremetz JF Garcia-Réguet N Conseil V Fourmaux MN. Apical Organelles and Host-Cell Invasion by Apicomplexa. Int J Parasitol (1998) 28(7):1007–13. 10.1016/S0020-7519(98)00076-9 9724870

[6] Asada M Goto Y Yahata K Yokoyama N Kawai S Inoue N. Gliding Motility of Babesia Bovis Merozoites Visualized by Time-Lapse Video Microscopy. PloS One (2012) 7(4):e35227. 10.1371/journal.pone.0035227 22506073 PMC3323635

[7] Rodriguez AE Florin-Christensen M Flores DA Echaide I Suarez CE Schnittger L. The Glycosylphosphatidylinositol-Anchored Protein Repertoire of Babesia Bovis and its Significance for Erythrocyte Invasion. Ticks Tick Borne Dis (2014) 5(3):343–8. 10.1016/j.ttbdis.2013.12.011 24642346

[8] Carcy B Précigout E Schetters T Gorenflot A. Genetic Basis for GPI-anchor Merozoite Surface Antigen Polymorphism of Babesia and Resulting Antigenic Diversity. Vet Parasitol (2006) 138(1-2):33–49. 10.1016/j.vetpar.2006.01.038 16551492

[9] Suarez CE Florin-Christensen M Hines SA Palmer GH Brown WC McElwain TF. Characterization of Allelic Variation in the Babesia Bovis Merozoite Surface Antigen 1 (MSA-1) Locus and Identification of a Cross-Reactive Inhibition-Sensitive MSA-1 Epitope. Infect Immun (2000) 6812:6865–70. 10.1128/IAI.68.12.6865-6870.2000 PMC97791 11083806

[10] Yang YS Murciano B Moubri K Cibrelus P Schetters T Gorenflot A. Structural and Functional Characterization of Bc28.1, Major Erythrocyte-Binding Protein From Babesia Canis Merozoite Surface. J Biol Chem (2012) 287(12):9495–508. 10.1074/jbc.M111.260745 PMC3308747 22294693

[11] Delbecq S Precigout E Vallet A Carcy B Schetters TP Gorenflot A. Babesia Divergens: Cloning and Biochemical Characterization of Bd37. Parasitology (2002) 125(Pt 4):305–12. 10.1017/S0031182002002160 12403318

[12] Cowman AF Crabb BS. Invasion of Red Blood Cells by Malaria Parasites. Cell (2006) 124(4):755–66. 10.1016/j.cell.2006.02.006 16497586

[13] Delbecq S Auguin D Yang YS Löhr F Arold S Schetters T. The Solution Structure of the Adhesion Protein Bd37 From Babesia Divergens Reveals Structural Homology With Eukaryotic Proteins Involved in Membrane Trafficking. J Mol Biol (2008) 375(2):409–24. 10.1016/j.jmb.2007.08.019 18035372

[14] Rodríguez AE Couto A Echaide I Schnittger L Florin-Christensen M. Babesia Bovis Contains an Abundant Parasite-Specific Protein-Free Glycerophosphatidylinositol and the Genes Predicted for its Assembly. Vet Parasitol (2010) 167(2-4):227–35. 10.1016/j.vetpar.2009.09.024 19833438

[15] Dominguez M Echaide I Echaide ST Mosqueda J Cetrá B Suarez CE. In Silico Predicted Conserved B-cell Epitopes in the Merozoite Surface Antigen-2 Family of B. Bovis are Neutralization Sensitive. Vet Parasitol (2010) 167(2-4):216–26. 10.1016/j.vetpar.2009.09.023 19850413

[16] Flores DA Rodriguez AE Tomazic ML Torioni de Echaide S Echaide I Zamorano P. Schnittger L Et Al: Characterization of GASA-1, a New Vaccine Candidate Antigen of Babesia Bovis. Vet Parasitol (2020) 287:109275. 10.1016/j.vetpar.2020.109275 33091630

[17] He L Feng HH Zhang WJ Zhang QL Fang R Wang LX. Occurrence of Theileria and Babesia Species in Water Buffalo (Bubalus Babalis, Linnaeus, 1758) in the Hubei Province, South China. Vet Parasitol (2012) 186(3-4):490–6. 10.1016/j.vetpar.2011.11.021 22154255

[18] Liu Z Ma L Zhao J. An Investigation of Water Buffalo Babesiosis in Hubei Province V. Adult Rhipicephalus Haemaphysaloides Transmits the Parasites Transovarially. Chin J Vet Sci (1989) 1:61–70. (in chinese).

[19] Nie Z Xia Y Yu L Li M Guo J Sun Y. An X Et Al: Characterization of the Variable Merozoite Surface Antigen (VMSA) Gene Family of Babesia Orientalis. Parasitol Res (2020) 119(11):3639–48. 10.1007/s00436-020-06877-z 32930858

[20] Berens SJ Brayton KA Molloy JB Bock RE Lew AE McElwain TF. Merozoite Surface Antigen 2 Proteins of Babesia Bovis Vaccine Breakthrough Isolates Contain a Unique Hypervariable Region Composed of Degenerate Repeats. Infect Immun (2005) 73(11):7180–9. 10.1128/IAI.73.11.7180-7189.2005 PMC1273907 16239512

[21] LeRoith T Berens SJ Brayton KA Hines SA Brown WC Norimine J. The Babesia Bovis Merozoite Surface Antigen 1 Hypervariable Region Induces Surface-Reactive Antibodies That Block Merozoite Invasion. Infect Immun (2006) 74(6):3663–7. 10.1128/IAI.00032-06 PMC1479293 16714599

[22] Singh SK Singh AP Pandey S Yazdani SS Chitnis CE Sharma A. Definition of Structural Elements in Plasmodium Vivax and P. Knowlesi Duffy-binding Domains Necessary for Erythrocyte Invasion. Biochem J (2003) 374(Pt 1):193–8. 10.1042/bj20030622 PMC1223586 12775212

[23] He L He P Luo X Li M Yu L Guo J. The MEP Pathway in Babesia Orientalis Apicoplast, a Potential Target for Anti-Babesiosis Drug Development. Parasit Vectors (2018) 11(1):452. 10.1186/s13071-018-3038-7 30081952 PMC6090808

[24] Zhan X He J Yu L Liu Q Sun Y Nie Z. Luo X Et Al: Identification of a Novel Thrombospondin-Related Anonymous Protein (BoTRAP2) From Babesia Orientalis. Parasite Vector (2019) 12(1):200. 10.1186/s13071-019-3457-0 PMC6500065 31053087

[25] Nikodem D Davidson E. Identification of a Novel Antigenic Domain of Plasmodium Falciparum Merozoite Surface Protein-1 That Specifically Binds to Human Erythrocytes and Inhibits Parasite Invasion, In Vitro. Mol Biochem Parasitol (2000) 108(1):79–91. 10.1016/S0166-6851(00)00206-1 10802320

[26] Wu M Zhao D Zhong W Yan H Wang X Liang Z. High-Density Distributed Electrode Network, a Multi-Functional Electroporation Method for Delivery of Molecules of Different Sizes. Sci Rep (2013) 3:3370. 10.1038/srep03370 24284649 PMC3842547

[27] Tattiyapong M Sivakumar T Takemae H Simking P Jittapalapong S Igarashi I. Genetic Diversity and Antigenicity Variation of Babesia Bovis Merozoite Surface Antigen-1 (MSA-1) in Thailand. Infection Genet evolution: Infect Genet Evol (2016) 41:255–61. 10.1016/j.meegid.2016.04.021 27101782

[28] Shu X Guo J Nie Z Xia Y He L Zhao J. A Novel 53 kDa Protein (BoP53) in Babesia Orientalis Poses the Immunoreactivity Using the Infection Serum. Parasitol Int (2020) 78:102152. 10.1016/j.parint.2020.102152 32512049

[29] Tian Y Li F Guo J Hu Y Shu X Xia Y. Nie Z Et Al: Identification and Characterizations of a Rhoptries Neck Protein 5 (BoRON5) in Babesia Orientalis. Parasitol Int (2020) 77:102106. 10.1016/j.parint.2020.102106 32179136

[30] Guo J Li M Sun Y Yu L He P Nie Z. Wang S Et Al: Characterization of a Novel Secretory Spherical Body Protein in Babesia Orientalis and Babesia Orientalis-Infected Erythrocytes. Parasite Vector (2018) 11(1):433. 10.1186/s13071-018-3018-y PMC6060518 30045776

[31] Ejigiri I Ragheb DR Pino P Coppi A Bennett BL Soldati-Favre D. Shedding of TRAP by a Rhomboid Protease From the Malaria Sporozoite Surface is Essential for Gliding Motility and Sporozoite Infectivity. PloS Pathog (2012) 8(7):e1002725. 10.1371/journal.ppat.1002725 22911675 PMC3406075

[32] Lin CS Uboldi AD Epp C Bujard H Tsuboi T Czabotar PE. Multiple Plasmodium Falciparum Merozoite Surface Protein 1 Complexes Mediate Merozoite Binding to Human Erythrocytes. J Biol Chem (2016) 291(14):7703–15. 10.1074/jbc.M115.698282 PMC4817195 26823464

[33] Dluzewski AR Ling IT Hopkins JM Grainger M Margos G Mitchell GH. Formation of the Food Vacuole in Plasmodium Falciparum: A Potential Role for the 19 kDa Fragment of Merozoite Surface Protein 1 (MSP1(19)). PloS One (2008) 3(8):e3085. 10.1371/journal.pone.0003085 18769730 PMC2518119

[34] Woehlbier U Epp C Hackett F Blackman MJ Bujard H. Antibodies Against Multiple Merozoite Surface Antigens of the Human Malaria Parasite Plasmodium Falciparum Inhibit Parasite Maturation and Red Blood Cell Invasion. Malar J (2010) 9:77. 10.1186/1475-2875-9-77 20298576 PMC2847572

[35] Boyle MJ Langer C Chan JA Hodder AN Coppel RL Anders RF. Sequential Processing of Merozoite Surface Proteins During and After Erythrocyte Invasion by Plasmodium Falciparum. Infect Immun (2014) 82(3):924–36. 10.1128/IAI.00866-13 PMC3958018 24218484

[36] Beeson JG Drew DR Boyle MJ Feng G Fowkes FJ Richards JS. Merozoite Surface Proteins in Red Blood Cell Invasion, Immunity and Vaccines Against Malaria. FEMS Microbiol Rev (2016) 40(3):343–72. 10.1093/femsre/fuw001 PMC4852283 26833236

[37] Shen B Buguliskis JS Lee TD Sibley LD. Functional Analysis of Rhomboid Proteases During Toxoplasma Invasion. mBio (2014) 5(5):e01795–01714. 10.1128/mBio.01795-14 PMC4212836 25336455

[38] Montero E Gonzalez LM Rodriguez M Oksov Y Blackman MJ Lobo CA. A Conserved Subtilisin Protease Identified in Babesia Divergens Merozoites. J Biol Chem (2006) 281(47):35717–26. 10.1074/jbc.M604344200 16982617

[39] Gallenti R Poklepovich T Florin-Christensen M Schnittger L. The Repertoire of Serine Rhomboid Proteases of Piroplasmids of Importance to Animal and Human Health. Int J Parasitol (2021) 68(12):6865–70. 10.1016/j.ijpara.2020.10.010 33610524

