# Supplementary figures and images for: Erythrocyte Adhesion of Merozoite Surface Antigen 2c1 Expressed During Extracellular Stages of Babesia orientalis
Source: Front Immunol. 2021 May 17;12:623492. doi: 10.3389/fimmu.2021.623492 (PMC8165267; doi:10.3389/fimmu.2021.623492)

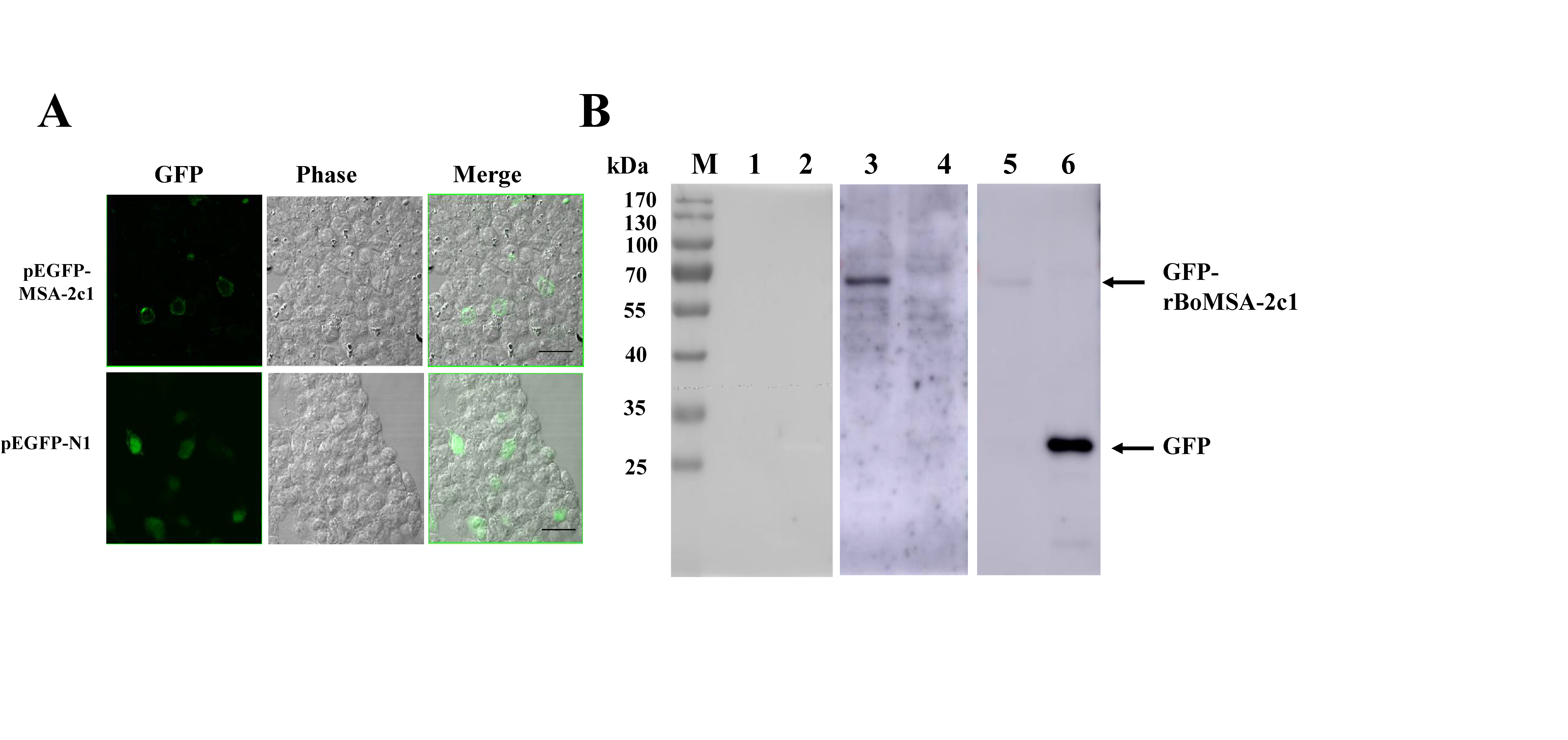

Supplement: Supplementary Figure 1 — Expression of BoMSA-2c1 on the HEK 293T cell surface. (A) Expression of green fluorescent protein (GFP) detected by scanning confocal microscopy (LSCM). BoMSA-2c1-GFP was located on the surface of HEK 293T cells transfected with pEGFP-BoMSA2c1-N1, and GFP was detected in the cytoplasm of HEK 293T cells, transfected with the control pEGFP-N1 vector. (B) Western blots of BoMSA-2c1 in HEK 293T cells. Lysates of HEK 293T cells transfected with pEGFP-BoMSA-2c1 (lanes 1, 3, and 5) and empty pEGFP-NI vector (lanes 2, 4, and 6). Lysates of negative control HEK 293T cells exhibiting reactions with PcAb-BoMSA-2c1 (lanes 3 and 4), anti-GFP (lanes 5 and 6), and pre-immune serum (lanes 1 and 2). Scale bars: 40 μm. [file Image_1.tif]
